# Cost-effectiveness of an enhanced Paramedic Acute Stroke Treatment Assessment (PASTA) during emergency stroke care: Economic results from a pragmatic cluster randomized trial

**DOI:** 10.1177/17474930211006302

**Published:** 2021-04-07

**Authors:** Nawaraj Bhattarai, Christopher I Price, Peter McMeekin, Mehdi Javanbakht, Luke Vale, Gary A Ford, Lisa Shaw

**Affiliations:** 1 Health Economics Group, Population Health Sciences Institute, 5994 Newcastle University, Newcastle upon Tyne, UK; 2 Stroke Research Group, Population Health Sciences Institute, 5994 Newcastle University, Newcastle upon Tyne, UK; 3 Faculty of Health & Life Sciences, Northumbria University, Newcastle upon Tyne, UK; 4 Medical Sciences Division, University of Oxford, and Oxford University Hospitals NHS Foundation Trust, Oxford, UK

**Keywords:** Stroke, cost-effectiveness, cluster randomized controlled trial, thrombolysis, paramedic, ambulance

## Abstract

**Background:**

The Paramedic Acute Stroke Treatment Assessment (PASTA) trial evaluated an enhanced emergency care pathway which aimed to facilitate thrombolysis in hospital. A pre-planned health economic evaluation was included. The main results showed no statistical evidence of a difference in either thrombolysis volume (primary outcome) or 90-day dependency. However, counter-intuitive findings were observed with the intervention group showing fewer thrombolysis treatments but less dependency.

**Aims:**

Cost-effectiveness of the PASTA intervention was examined relative to standard care.

**Methods:**

A within trial cost-utility analysis estimated mean costs and quality-adjusted life years over 90 days’ time horizon. Costs were derived from resource utilization data for individual trial participants. Quality-adjusted life years were calculated by mapping modified Rankin scale scores to EQ-5D-3L utility tariffs. A post-hoc subgroup analysis examined cost-effectiveness when trial hospitals were divided into compliant and non-compliant with recommendations for a stroke specialist thrombolysis rota.

**Results:**

The trial enrolled 1214 patients: 500 PASTA and 714 standard care. There was no evidence of a quality-adjusted life year difference between groups [0·007 (95% CI: −0·003 to 0·018)] but costs were lower in the PASTA group [−£1473 (95% CI: −£2736 to −£219)]. There was over 97.5% chance that the PASTA pathway would be considered cost-effective. There was no evidence of a difference in costs at seven thrombolysis rota compliant hospitals but costs at eight non-complaint hospitals costs were lower in PASTA with more dominant cost-effectiveness.

**Conclusions:**

Analyses indicate that the PASTA pathway may be considered cost-effective, particularly if deployed in areas where stroke specialist availability is limited.

**Trial Registration:** ISRCTN12418919 www.isrctn.com/ISRCTN12418919

## Introduction

Intravenous thrombolysis for ischaemic stroke is a cost-effective treatment, but large variations in provision exist.^1,2^ Previous studies have described improvements in the volume and/or speed of treatment following the introduction of ambulance pre-notification,^3^ multidisciplinary training^4^ and a higher priority response for suspected stroke,^5^ but none have reported the economic impact of a pre-hospital intervention intended to promote thrombolysis delivery.

The Paramedic Acute Stroke Treatment Assessment (PASTA) multicenter cluster randomized controlled trial examined whether an enhanced paramedic emergency stroke assessment pathway for patients presenting within 4 hours of stroke onset could improve thrombolysis volume (primary outcome) when compared to standard care (SC).^6,7^ Secondary outcomes included dependency at day 90 after stroke (modified Rankin Score (mRS)) and resource utilization data for a pre-planned health economic analysis. The PASTA intervention comprised additional prehospital information collection, a structured hospital handover, practical assistance after handover, a pre-departure care checklist, and clinician feedback.

Although there was no statistical evidence of a difference between the trial groups for the proportion of patients who received thrombolysis (primary outcome), contrary to the anticipated effect of the intervention, less people received treatment in the PASTA group [PASTA: 197/500 (39.4%) versus SC: 319/714 (44.7%); adjusted Odds Ratio (aOR) 0·81 (95% CI: 0·61–1·08); *p* = 0·15].^7^ There was also no statistical evidence of a difference between the trial groups in dependency at day 90 after stroke (modified Rankin Score (mRS)) grades 3–6); however, counter-intuitive to the lower thrombolysis rate, fewer patients were dependent in the PASTA group [PASTA: 313/489 (64.0%) versus SC: 461/690 (66.8%); aOR 0.86 (95% CI: 0.60–1.20); *p* = 0.39]. These unexpected findings led to a post-hoc analysis to explore how stroke specialist availability impacted upon thrombolysis treatment. At 8/15 trial hospitals that were not fully compliant with a national recommendation for specialist input into all thrombolysis decisions, there was a significant 9.8% reduction in thrombolysis in the PASTA group compared to SC [99/276 (35.9%) PASTA versus 105/230 (45.7%) SC; unadjusted OR 0·67 (95% CI: 0·47–0·95); *p* = 0·03]. Whereas for the 7/15 hospitals that were compliant, there was no evidence of a difference in thrombolysis rates [98/224 (43.8%) PASTA vs. 214/484 (44·2%) SC; unadjusted OR 0·98 (95% CI: 0·71–1·35); *p* = 0·91]. We proposed a hypothesis that structured handover of additional information and/or a multidisciplinary checklist improved the selection of patients for thrombolysis, particularly in hospitals with reduced specialist availability.^7^ Cost-effectiveness results showing a similar pattern would be consistent with this theory.

## Aim

This manuscript reports the pre-planned cost-effectiveness analysis of the PASTA intervention and analyses for the two post-hoc subgroups defined by local specialist availability.

## Methods

### PASTA trial design summary

The PASTA trial protocol is reported elsewhere.^6,7^ In summary, a pragmatic multicenter cluster randomized controlled trial was hosted by three UK ambulance services (North East, North West and Wales) and 15 Hyperacute Stroke Units (HASUs). Clusters were individual paramedics based within ambulance stations pre-randomized to PASTA training or continuing SC. Paramedics at PASTA stations had to successfully complete training prior to their involvement in the trial (accessed online). Paramedics at SC stations were advised that their routinely recorded clinical data would be used in a research study.

Patients were identified and recruited to the trial by hospital staff after completion of the thrombolysis assessment in participating HASUs. Eligible patients were those where a hospital specialist confirmed a stroke diagnosis and a study paramedic had attended within 4 h of symptom onset. Written consent was obtained. The primary outcome was the proportion of patients receiving thrombolysis. Secondary outcomes included key time intervals across the emergency stroke pathway and day 90 mRS. The study sample size calculation was 1149 participants which provided 90% power to detect a change from 43% to 53% of study eligible patients receiving thrombolysis.

The National Research Ethics Committee North East – Newcastle and North Tyneside 1 (reference 15/NE/0309) approved the study.

### Resource use and costs

Resource use data for each patient was collected using case report forms and questionnaires. Resource use included PASTA pathway training time (PASTA group only), ambulance time from ‘on-scene’ to ‘clear’, acute assessments and treatments, length of stay in hospital, post-discharge rehabilitation, social services involvement (paid carers at home and in social care settings) and hospital readmissions. Unit costs were derived from routine sources for the NHS and social care,^8,9^ and other published sources. Details are reported in Table S1 and S2, supplementary material. Where necessary, the unit costs were inflated to 2017/2018 costs using the Hospital and Community Health Services (HCHS) pay and price inflation indices.^10^ Costs are presented in UK Sterling Pounds. The total cost for each participant was calculated as the sum of a number of cost components (e.g. ambulance time, inpatient care cost, social care cost).

### Utilities and QALYs

Utility values were generated by mapping day 90 mRS scores to EQ-5D-3 L values^11^ using previously reported algorithms.^11,12^ Where there were missing 90-day mRS scores, routinely captured discharge mRS scores were carried forward. Deceased patients received a mRS value of 6. The utility values were then combined with length of life over the trial follow-up to estimate QALYs for each participant using the area under the curve method.^13^

### Economic evaluation

A cost-utility analysis was undertaken to compare costs and quality adjusted life years (QALYs) between PASTA and SC.^14^ Cost-effectiveness was expressed as incremental costs per QALY gained. The analysis took the perspective of UK NHS and personal social services. As the trial duration of 90 days was the time horizon for the economic analysis, discounting of costs and outcomes was not required. Resource use, cost and QALY data were analyzed using STATA v14·2.

The base-case cost-effectiveness analysis was carried out using the complete case data. Generalized linear model regressions with gamma family link function estimated marginal costs and QALY gains whilst controlling for age, sex and baseline (pre-stroke) utility clustered by site.^13^ Non parametric bootstrapping^15^ with 1000 bootstraps was used on the costs and QALYs to estimate the mean difference in costs and QALYs and their 95% CI between PASTA pathway and standard care to quantify the degree of uncertainty. Additional analyses without baseline covariate adjustments or bootstrap were carried out using both complete case and available case data to check how the mean differences in costs and QALYs differed from the base-case estimates.

#### Sensitivity analysis

Stochastic sensitivity analysis, which used the non-parametric bootstrapping technique^15^ with 1000 bootstraps as described earlier, was used to explore the impact of statistical imprecision surrounding the point estimates of costs, QALYs and cost-effectiveness. A cost-effectiveness acceptability curve (CEAC)^16^ was generated using the bootstrapped estimates of incremental costs and QALYs to illustrate uncertainty surrounding the cost-effectiveness estimate. The CEAC demonstrates the probability of each care pathway being cost-effective over a range of willingness to pay values. A cost-effectiveness (CE) plane (scatterplot) was also generated to visualize the uncertainties in point estimates of incremental costs and QALYs.

Further sensitivity analyses were conducted to assess the impact of uncertainties surrounding a number of assumptions made in the cost-effectiveness analysis, notably the changes in utility estimates and use of imputed data. As the base-case cost-effectiveness analysis utilized algorithms for utility values from Whynes et al.^12^ to estimate QALYs, the impact of using alternative algorithms from Rivero-Arias et al.^11^ was assessed. A second analysis used imputed data where any missing total cost (considered missing if any cost component was missing) and utility data were imputed using predictive mean matching (PMM) within the multiple imputation generated using chained equations.^17^

#### Subgroup analysis

Participating hospitals were categorized as compliant or non-compliant with UK recommendations for provision of a specialist thrombolysis on call rota using workforce information available in the National Sentinel Stroke Audit Programme Acute Organizational Audit 2016.^2^ Compliance was defined as a minimum of six specialists trained in emergency stroke care providing a continuous rota without input from non-specialists, so that all treatment decisions are made by a stroke specialist from the same service either in person or via telemedicine.^18^ Costs, QALYS, and cost-effectiveness were calculated as described above for the base-case analysis for each subgroup, i.e. patients at complaint hospitals (*n* = 7) and patients at non-compliant hospitals (*n* = 8).

## Results

From 121 ambulance stations randomized for the trial, 453/817 paramedics from 62 PASTA stations completed training to participate and 700/723 from 59 SC stations agreed to involvement. During the trial enrolment period, 11,478 stroke patients conveyed by ambulance were screened by participating HASU staff, 1391 were eligible and approached about enrolment, and 1214 gave consent to take part. Of the 1214 enrolled patients, 500 were assessed by 242 PASTA trained paramedics (2.1 patients per paramedic) and 714 were assessed by 355 SC paramedics (2.0 patients per paramedic).^7^ Demographic and clinical characteristics were similar in both groups and are reported elsewhere.^7^

The base-case cost-effectiveness analysis (complete case data) showed no evidence of a difference in QALYs between the groups [PASTA: 0.108 (95% CI: 0.099–0.116); SC: 0.100 (95% CI 0.093–0.108); incremental QALY: 0·007 (95% CI −0·003 to 0·018)] over the 90-day follow-up period; however, total costs were significantly lower in the PASTA group [incremental cost: −£1,473 (95% CI −£2736 to −£219)] (Table 1). When complete case data were analyzed without baseline adjustment or bootstrapping, a similar pattern of lower costs in the PASTA group but no evidence of a difference in QALYS was seen (Table S3).

**Table 1.** Cost-effectiveness of PASTA pathway versus Standard Care.

Scenario	Intervention strategy	Total Cost (£) [Mean (95% CI)]	QALY [Mean (95% CI)]	Difference in cost [Mean (95% CI)]	Difference in QALY [Mean (95% CI)]	ICER	Probability of PASTA care pathway being considered cost-effective at different threshold values for society’s WTP per QALY gain			
							*£0*	*£20 k*	*£30 k*	*£50 k*
*Base-case analysis*										
Complete case	PASTA (*N* = 394) SC (*N* = 556)	11,630 (10,702–12,586) 13,103 (12,292–14,019)	0.108 (0.099–0.116) 0.100 (0.093–0.108)	−1473 (−2736 to −219)	0.007 (−0.003 to 0.018)	PASTA Dominant	98.6%	99%	99.2%	99.2%
*Sensitivity analysis*										
Alternative utility algorithm	PASTA (*N* = 394) SC (*N* = 556)	11,647 (10,770–12,570) 13,094 (12,219–13,958)	0.108 (0.1–0.117) 0.1 (0.093 to 0.108)	−1448 (−2689 to −122)	0.008 (−0.002 to 0.018)	PASTA Dominant	97.9%	98.6%	98.8%	99.2%
Imputed dataset	PASTA (*N* = 500) SC (*N* = 714)	12,019 (11,223–12,865) 13,106 (12,421–13,904)	0.109 (0.102–0.117) 0.104 (0.097–0.110)	−1086 (−2236 to −13)	0.005 (−0.004 to 0.015)	PASTA Dominant	97.6%	98.1%	98.3%	98.3%
*Subgroup analysis*										
Hospitals compliant with thrombolysis rota guidelines	PASTA (*N* = 168) SC (*N* = 371)	12,119 (10,629–13,512) 12,542 (11,528–13,603)	0.103 (0.091–0.115) 0.098 (0.089–0.107)	−423 (−2220 to 1362)	0.005 (−0.008 to 0.018)	PASTA Dominant	66.6%	70.5%	71.5%	73.8%
Hospitals non- compliant with thrombolysis rota guidelines	PASTA (*N* = 226) SC (*N* = 185)	11,262 (10,053–12,579) 14,213 (12,747–15,713)	0.112 (0.100–0.124) 0.103 (0.090–0.115)	−2952 (−4988 to −917)	0.009 (−0.008 to 0.025)	PASTA Dominant	99.9%	99.9%	99.9%	99.9%

Note: Results are bootstrapped regressed estimates; *N* = Number of participants observed; Difference estimates: PASTA minus SC.

Breakdowns of resource utilization and costs (available case data) are shown in Tables S4 and S5, respectively (see supplementary file). This indicates that lower costs in the PASTA group were in part driven by the lower costs of index hospital admissions (∼£440) and acute treatment costs, predominantly due to fewer thrombolysis treatments (∼£300), but there was also a post-discharge saving due to lower requirements for community rehabilitation and care homes (∼£470). A QALY breakdown is shown in Table S6.

### Sensitivity analysis

**Figure 1.**
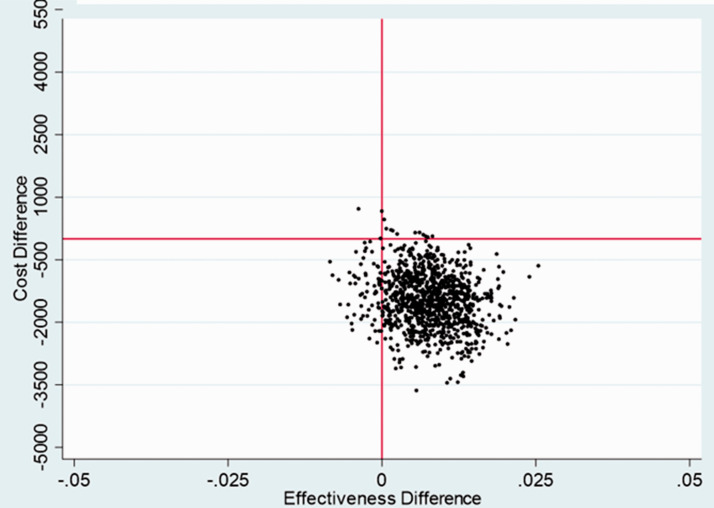
Cost-effectiveness plane—base case analysis.

**Figure 2.**
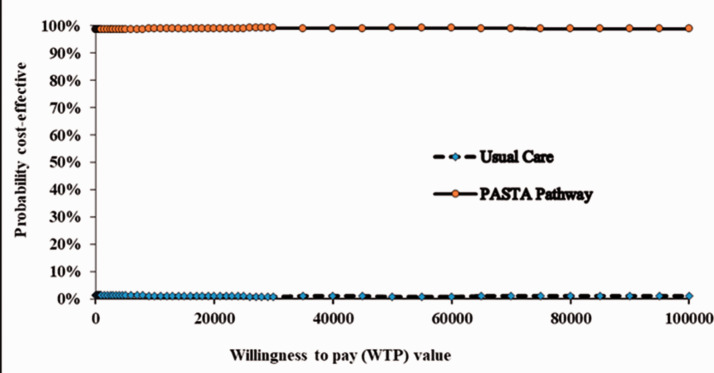
Cost effectiveness acceptability curve—base case analysis.

A plot of bootstrapped incremental costs and QALYs showed the uncertainties in point estimates of incremental costs and QALYs in the base-case analysis, and for a majority of iterations the PASTA group was less costly and more effective (i.e. dominant over SC) (Figure 1). Furthermore, over the plausible range of values for society’s willingness to pay for a QALY, there was over a 97.5% chance that PASTA would be considered cost-effective (Figure 2; Table 1).

Further sensitivity analyses showed that the base-case results were in general robust to changes in parameter assumptions including the alternative utility algorithms, and imputation of missing cost and QALY data. The PASTA pathway still had over 97.5% probability of being considered cost-effective over the range of willingness to pay values for these sensitivity analyses (Table 1).

### Subgroup analysis

**Figure 3.**
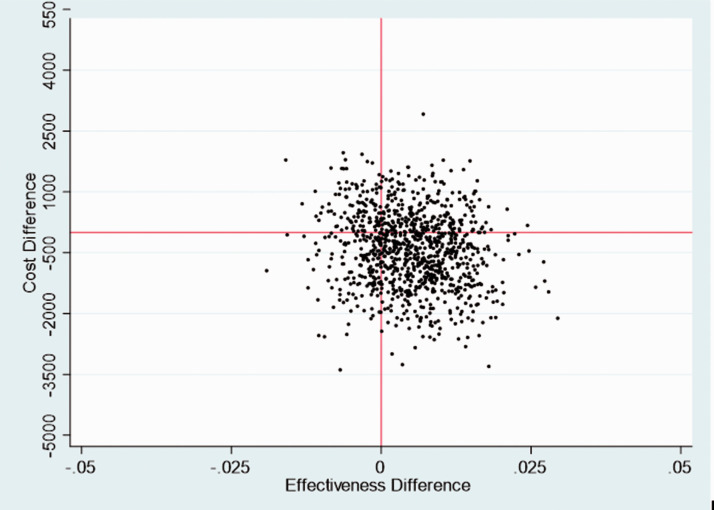
Cost-effectiveness plane: compliant hospitals.

**Figure 4.**
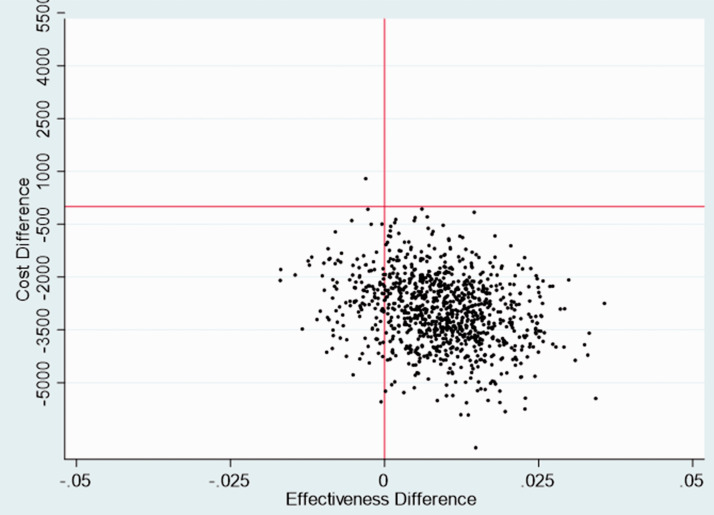
Cost-effectiveness plane: non-compliant hospitals.

Thrombolysis guideline compliant and non-compliant hospital results are reported in Table 1. There was no evidence of a difference in costs [−423 (95% CI−2,220 to 1362)] or in QALYs [0.005 (95% CI−0.008 to 0.018)] for those seven hospitals compliant with the thrombolysis guidelines. There was less than 74% probability that the PASTA pathway would be considered cost-effective over the range of willingness to pay values. However, in the eight non-compliant hospitals, the costs were significantly lower in the PASTA group [−2,952 (95% CI−4,988 to −917)] and there was a 99% probability that the PASTA pathway would be considered cost-effective. Uncertainties in the point estimates of incremental costs and QALYs in each subgroup are visualized in their respective CE plots (Figures 3 and 4).

## Discussion

This economic evaluation has shown that the PASTA trial group had lower costs than standard care and when costs were considered alongside data on QALY difference, there was a very high chance that the PASTA intervention would be cost-effective across all threshold values for society’s willingness to pay. This finding was consistent across all sensitivity analyses. The subgroup analyses indicated that cost-effectiveness was particularly likely across services with specialist availability below the level recommended by national guidelines.

Whilst the lower costs were in part related to fewer thrombolysis treatments in the PASTA group, there were also savings observed in other aspects of care including length of stay, rehabilitation and social care. These latter findings are consistent with the direction of the QALY difference between the groups, as patients with better health would require lower costs for these resources.^19^ It is surprising, however, that patients were generally in better health and incurred fewer care costs in the PASTA group when there was no statistical evidence of a difference in thrombolysis rate (primary outcome) and indeed fewer thrombolysis treatments were observed.

We have previously hypothesized that the counter-intuitive main trial observations of a lower thrombolysis rate and better health outcomes in the PASTA group may be explained by a theory that the PASTA intervention led to greater caution during patient selection for thrombolysis when the benefit to risk ratio was borderline and thereby this avoided futile treatment and lowered the risk of harm from adverse events.^7^ Other aspects of acute care might also have been performed better amongst the intervention group if PASTA generally reinforced adherence to acute care guidelines. As the lower thrombolysis rate was particularly evident across services with specialist availability below the level recommended by national guidelines, the relative inexperience of non-specialists may routinely lead to over-rather than under-treatment of borderline cases when weighing up complex information under time pressure. Such behavior may have been moderated by the more detailed and structured content of the enhanced paramedic assessment including details about bleeding risk (e.g. recent surgery and anticoagulant medication) and pre-stroke dependency.^7^ In non-stroke specialties, there is already evidence that simple tools to structure paramedic to ED handover^20,21^ and multidisciplinary care process checklists^22,23^ can standardize communication of key information and improve the quality of care. As the trial findings were unexpected, we did not collect detailed information about individual treatment decisions and additional interventions which would be needed to confirm our theory; however, we believe that both the main and post-hoc health economic analyses are consistent with and provide support to this suggestion.

Interpretation of trials with a neutral primary outcome yet dominant cost-effectiveness is variable with some authors reporting that the intervention should be adopted but others stating uncertainty or favoring the control.^24^ The PASTA trial is further complicated when the counter-intuitive nature of the main trial observations are considered. The results of the health economic analyses could of course be due to chance as the study was not powered to detect differences in costs, QALYs, and cost-effectiveness. However, as PASTA was a large trial across multiple hospital sites and confidence intervals for costs and QALYs were relatively narrow, there is a very high likelihood that the PASTA intervention would be considered cost-effective even though the underlying mechanism requires further investigation. As cost-effectiveness was shown for >97.5% of willingness to pay scenarios, this is analogous to a one-sided *p* value <0.025 that cost-effectiveness would be acceptable.

The main strength of our study was the use of a randomized controlled cluster design involving large numbers of patients across multiple HASUs operating under the same National Clinical Guidelines and costing frameworks. The main limitation of the economic analysis is that utility values were estimated using published algorithms for mapping mRS scores on to the EQ-5D rather than being based on responses to the EQ-5D collected directly from participants. However, the algorithm used has been well validated and it is reassuring that conclusions did not change when an alternative utility algorithm was applied. Although the QALY difference found was small and therefore potentially prone to measurement error, the value reflects the entire trial population whereas only a proportion of patients received thrombolysis, which itself is a treatment that only benefits or harms a proportion of those who are treated. Consequently, it may not be surprising that the QALY difference found was small. In addition, follow-up was short term at 90 days whereas QALY gain may be greater over a longer period as patients in better health by day 90 are likely to be those with a changed recovery trajectory which would translate into additional further gain over time.^25,26^ It should also be acknowledged that the study took a UK personal social care perspective, and the findings may not apply in other healthcare settings.

This is the first study to formally evaluate the 90-day cost-effectiveness of a paramedic-led process to improve outcomes during emergency care of stroke patients and illustrates the importance of considering economic consequences of complex interventions. Further investigation is required to understand the specific effects upon clinical decisions and care delivery, but our data indicate that the PASTA pathway is likely to be a cost-effective intervention, particularly if it is deployed in areas where hospital stroke specialist availability is limited.

## Supplemental Material

sj-pdf-1-wso-10.1177_17474930211006302 - Supplemental material for Cost-effectiveness of an enhanced Paramedic Acute Stroke Treatment Assessment (PASTA) during emergency stroke care: Economic results from a pragmatic cluster randomized trial Click here for additional data file. Supplemental material, sj-pdf-1-wso-10.1177_17474930211006302 for Cost-effectiveness of an enhanced Paramedic Acute Stroke Treatment Assessment (PASTA) during emergency stroke care: Economic results from a pragmatic cluster randomized trial by Nawaraj Bhattarai, Christopher I Price, Peter McMeekin, Mehdi Javanbakht, Luke Vale, Gary A Ford and Lisa Shaw in International Journal of Stroke
