# Supplementary material for: Cost-effectiveness of an enhanced Paramedic Acute Stroke Treatment Assessment (PASTA) during emergency stroke care: Economic results from a pragmatic cluster randomized trial
Source: Int J Stroke. 2021 Apr 7;17(3):282–90. doi: 10.1177/17474930211006302 (PMC8864331; doi:10.1177/17474930211006302)
Supplement: sj-pdf-1-wso-10.1177_17474930211006302 - Supplemental material for Cost-effectiveness of an enhanced Paramedic Acute Stroke Treatment Assessment (PASTA) during emergency stroke care: Economic results from a pragmatic cluster randomized trial [file sj-pdf-1-wso-10.1177_17474930211006302.pdf]

Cost-effectiveness of an enhanced Paramedic Acute Stroke Treatment Assessment (PASTA) during emergency stroke care: economic results from a pragmatic cluster randomised trial

Supplementary File

Table S1: Unit costs of resource item except early supported discharge

Cost Item	Unit cost	Source
**Ambulance (cost per minute)**	£3.76	NHS Reference cost 2017/18 (ASS02: See and treat and convey). The ambulance cost for per incident is £252 and per minute is given by dividing £252 by 67 min the assumed average ambulance time per incident.
**Paramedic training (cost per patient)**	£3.17	Ambulance Trusts; There are around 100,000 stroke cases each year in the UK (https://www.stroke.org.uk/system/files/sotn_2018.pdf). There are 24,400 qualified paramedics (https://digital.nhs.uk/data-and-information/publications/statistical/monthly-nhs-hospital-and-community-health-service-hchs-workforce-statistics). So an average 8.2 patients per paramedic (200,000/24,400) in 2 years was assumed. The hourly wage of paramedics is £26. Therefore, the mean paramedic training cost is £26/ 8.2
**Paramedic Training material productions (cost per patient)**	£0.03	The cost of developing training materials was assumed £6000 (PASTA Trial). Training materials could be used unlimited times for all the paramedics (24,400 paramedics) in the UK but are updated every two years. The average cost per paramedic is £0.245 (i.e. £6000/24,400). Given 8.2 patients per paramedic in 2 years the cost of training materials per patient was estimated as £0.03 (£0.245/8.2)
**MRI**	£138	NHS Reference cost 2017/18 (RD01A: Magnetic Resonance Imaging Scan of One Area, without Contrast, ≥19 years)
**CT scan**	£88.21	NHS Reference cost 2017/18 (RD02A: Computerised Tomography Scan of One Area, without Contrast, ≥19 years)
**Thrombolysis**	£5,670	NHS Reference cost 2017/18 YR23A-B (weighted average cost: Non-elective Long Stay)
**Neurosurgical assessment**	£208	NHS Reference cost 2017/18 (outpatients)
**Stroke unit cost per excess bed day**	£317	NHS Reference cost 2017/18 – average cost per day of non-elective excess bed days AA35A-F. Excess bed days cost was added to the cost where the length of stay in the study exceeded the weighted average length of stay in the HRG codes AA35A-F.
**Stroke unit cost per day**	£456	NHS Reference cost 2017/18 - average per day cost in non-elective long-stay stroke patient AA35A-F
**Intra-arterial (IA) treatments (mechanical thrombectomy)**	£8,111	https://www.gla.ac.uk/media/media_561299_en.pptx
**Blood pressure lowering prior to IV thrombolysis**	£12.27	British National Formulary; Intravenous Labetalol 50mg, dose to be given over at least 1 minute, then 50mg after 5 minutes if required.
**IV blood pressure lowering in haemorrhagic stroke**	£12.27	Assumed same as "Blood pressure lowering" above
**Reversal of causes of excessive bleeding**	£3.78+£1.89 +£151.8	£3.78 is the Cost of Konakion MM 10mg/1ml solution for injection ampoules (Roche products ltd).<br>£1.89 is the cost of %m by IV (in combination with Beriplex).<br>£151.80 is the cost of BeneFIX 250 unit powder and solvent for solution for infusion vials
**Referral for neurological assessment**	£208	NHS reference costs 2015/16 inflated to 2017/18

Cost Item	Unit cost	Source
**Transfer for neurological assessment**	£208+£252	NHS reference costs 2015/16 inflated to 2017/18; Includes transfer cost and neurological assessment costs
**Referral for Intra Arterial assessment**	£208	NHS reference costs 2015/16 inflated to 2017/2018
**Transfer for Intra Arterial Assessment**	£208+£252	NHS reference costs 2015/16 inflated to 2017/2018; Includes transfer costs and Intra-Arterial assessment costs
**Community rehabilitation per patient referred**	£2906.49	PSSRU 2014 inflated to 2017/18 prices
**Care home per day**	£164.64	PSSRU 2018 1.3 –not including personal expenses
**Paid care – community care package per week mRS=1 (1 hours/wk)**	£27.09	PSSRU 2018 8.1 – older person very low cost (av.per wk)
**Paid care – community care package per week mRS=2 (4 hours/wk)**	£108.37	PSSRU 2018 8.1 – older person low cost (av per wk)
**Paid care – community care package per week mRS=3-5 (10 hours/wk)**	£270.92	PSSRU 2018 8.1 – older person med-high cost (av per wk)
**Readmissions (cost depends on reasons for readmissions)**	Per readmission reason	NHS reference costs 2017/18. Mean costs were calculated using unit costs (derived using weighted average relevant HRG codes costs and length of stay) plus excess bed days costs if necessary e.g readmission with a stroke was the weighted average of costs of HRG code AA35A-F (non-elective long stay). The av length of hospital stay was 9.55 days and the weighted av cost was £4351. The av cost per excess bed days for stroke readmission was £317.

Table S2: Unit costs of Early supported discharge. *Source: Sentinel Stroke National Audit Programme (SSNAP) data*[1]

Age group	mRS	Mean number of Occupational therapy sessions	Mean number of Physiotherapy sessions	Mean number of Speech and language therapy sessions	Mean number of hours of psychotherapy	Mean costs with 23% overheads inflated to 2018 price
**40-74**	0	6.5	4.9	5.2	3.4	£1,833
**40-74**	1	6.6	5.3	5.8	2.7	£1,871
**40-74**	2	7.9	6.4	6.5	2.8	£2,165
**40-74**	3	10.2	8.2	7.5	2.5	£2,611
**40-74**	4	15.3	11.3	7.4	2.1	£3,288
**40-74**	5	12.6	9.9	7.8	1.9	£2,942
**75-100**	0	6.4	5.1	4.9	2	£1,684
**75-100**	1	6.5	5.1	5.1	2.2	£1,733
**75-100**	2	7.5	6.1	6	1.3	£1,927
**75-100**	3	8.7	7.6	6.6	1.8	£2,261
**75-100**	4	10.5	8	5.1	1.9	£2,313
**75-100**	5	9.2	7.4	3.8	1.7	£1,984

[1] Xu X-M, Vestesson E, Paley L, Desikan A, Wonderling D, Hoffman A, et al. The economic burden of stroke care in England, Wales and Northern Ireland: Using a national stroke register to estimate and report patient-level health economic outcomes in stroke. European Stroke Journal. 2018;3(1):82-91.

Table S3: Unadjusted mean mRS scores, utility, QALYs and total cost per participant

Measure	**PASTA** (N=394) [Mean (95% CI)]	**Standard care** (N=556) [Mean (95% CI)]	**Mean Difference** (95%CI**)**
mRS score			
Baseline (pre-stroke)	1.308 (1.160 to 1.456)	1.249 (1.126 to 1.371)	0.059 (-0.132 to 0.250)
90 days	3.279 (3.058 to 3.501)	3.426 (3.247 to 3.605)	0.147 (-0.429 to 0.135)
Utility value			
Baseline (pre-stroke)	0.754 (0.730 to 0.778)	0.762 (0.742 to 0.783)	-0.008 (-0.040 to 0.023)
90 days	0.430 (0.391 to 0.469)	0.409 (0.377 to 0.441)	0.021(-0.0285 to 0.0713)
QALYs	0.107 (0.097 to 0.116)	0.101 (0.093 to 0.108)	0.006(-0.006 to 0.0184)
Total cost	11607 (10672 to 12541)	13016 (12170 to 13862)	-1409 (-2684 to -135)

Note: Numbers are unadjusted and non-bootstrapped estimates from complete case data; Baseline: pre stroke; 90 days: post stroke; QALY: Quality Adjusted Life Years; mRS=Modified Rankin Scale; CI: Confidence Interval

Table S4: Resource use

**Resource**	**PASTA pathway**		**Standard care**	
Item	Observations	Resource use: Mean (SD)	Observations	Resource use: Mean (SD)
Ambulance time use minutes	433	80.4 (36.0)	612	67.8 (23.3)
Length of stay in hospital days	474	18.4 (24.6)	664	19.63 (25.6)
Item	Observations	Resource use: n (%)	Observations	Resource use: n (%)
Imaging	499		714	
CT scan		497 (99.60)		712 (99.72)
MRI		2 (0.40)		2 (0.28)
Thrombolysis	500	197 (39.4)	714	319 (44.68)
Thrombectomy	499	11 (2.2)	713	13 (1.8)
Other treatments	500		714	
Blood pressure lowering prior to IVT		37 (7.40)		47(6.58)
IV blood pressure lowering in haemorrhage		45 (9)		47 (6.58)
Reversal of abnormal coagulation in haemorrhage		14 (2.80)		16 (2.24)
Referral for neurological assessment		45 (9)		46 (6.44)
Transfer for neurological assessment		4 (0.80)		9 (1.26)
Referral for Intra Arterial assessment		13(2.60)		18(2.52)
Transfer for Intra Arterial Assessment		12 (2.40)		15 (2.10)
Discharge destination-care home	476	41 (8.61)	664	77 (11.60)
Paid care at private residence	468	61 (13.03)	657	73 (11.11)
Early supported discharge	470	69 (14.68)	662	157 (23.72)
Community rehabilitation	469	114 (24.31)	662	149 (22.51)
Number of Readmissions	468		659	
0		417 (89.10)		583 (88.47)
1		40 (8.55)		66 (10.06)
2		11 (2.35)		10 (1.52)

SD= Standarard deviation; n=number using resources

Table S5: Cost breakdown

	PASTA pathway					Standard Care				
**Items**	**N**	**Mean**	**SD**	**Min**	**Max**	**N**	**Mean**	**SD**	**Min**	**Max**
**Cost items**										
Cost of ambulance	433	£302.4	135.4	£106.8	£2,160.9	612	£255.2	87.8	£8.2	£649.1
Cost of paramedic training	500	£3.2	0.0	£3.2	£3.2	714	£0.0	0.0	£0.0	£0.0
Cost of hospital admission	474	£6,685.4	8,148.8	£456	£29,920.0	664	£7122.2	8,431.3	£456	£29,920.0
Cost of imaging	499	£88.4	3.2	£88.2	£138.0	714	£88.3	2.6	£88.2	£138.0
Cost of thrombolysis	500	£2,234.0	2,773.3	£0.0	£5,670.0	714	£2,533.2	2,820.9	£0.0	£5,670.0
Cost of Intra-arterial (IA) treatments	499	£178.8	1,192.1	£0.0	£8,111.0	713	£147.9	1,086.0	£0.0	£8,111.0
Cost of other treatments	500	£53.5	188.9	£0.0	£1,336.0	714	£45.0	178.6	£0.0	£1,336.0
Cost of care home	476	£616.4	2,487.2	£0.0	£14,817.6	664	£882	2,914.6	£0.0	£14,653.0
Cost of early supported discharge	470	£325.0	797.3	£0.0	£3,288.0	662	£541.4	993.2	£0.0	£3,288.0
Cost of community rehabilitation	469	£706.4	1,247.8	£0.0	£3288.0	662	£654.1	1,214.6	£0.0	£2,906.0
Paid carer visited at private residence	468	£244.5	737.6	£0.0	£3,444.5	657	£211.3	682.1	£0.0	£3,444.6
Cost of hospital re-admission	468	£570.55	2080.1	£0.0	£22061.0	659	£562.54	1956.82	£0.0	£16,456.0
Total cost	398	£11,808.89	9,603.08	£654.2	£41,476.7	562	£13,217.02	10,290.96	£692.1	£47,627.2
Difference in Total costs (PASTA minus Standard Care)	£ -1408 (95% CI : -2695 to -121)									

SD=Standard Deviation; N= participant sample; numbers are descriptive estimates based on available cases for each item.

Table S6: Number of days lived, utilities, and QALYs

	PASTA pathway					Standard Care				
	**N**	**Mean**	**SD**	**Min**	**Max**	**N**	**Mean**	**SD**	**Min**	**Max**
**Number of days lived**	493	68.72	35.40	0	90	697	68.55	35.45	0	90
**Utility values**										
Baseline (pre-stroke)	494	0.760	0.240	-0.15	0.93	708	0.768	0.241	-0.15	0.93
90 days	489	0.438	0.389	-0.15	0.93	690	0.421	0.379	-0.15	0.93
QALYs	489	0.109	0.094	-0.037	0.229	690	0.104	0.092	-0.037	0.229
Difference in QALYs (PASTA minus Standard Care)	0.005 (-0.006 to 0.016)									

SD=Standard Deviation; N= participant sample; QALY: Quality Adjusted Life Years; numbers are descriptive estimates based on available cases for each item.
